# Establishing a clinical informatics umbilical cord: lessons learned in launching infrastructure to support dyadic mother/infant primary care

**DOI:** 10.1093/jamiaopen/ooad065

**Published:** 2023-08-18

**Authors:** Seuli Bose-Brill, Rachel D’Amico, Adam Bartley, Robert Ashmead, Paola Flores-Beamon, Shadia Jallaq, Kevin Li, Shengyi Mao, Shannon Gillespie, Naleef Fareed, Kartik K Venkatesh, Norah L Crossnohere, Jody Davis, Alicia C Bunger, Allison Lorenz

**Affiliations:** Division of General Internal Medicine, Department of Internal Medicine, The Ohio State University College of Medicine, Columbus, Ohio, USA; Division of General Internal Medicine, Department of Internal Medicine, The Ohio State University College of Medicine, Columbus, Ohio, USA; Ohio Colleges of Medicine Government Resource Center, The Ohio State University, Columbus, Ohio, USA; Ohio Colleges of Medicine Government Resource Center, The Ohio State University, Columbus, Ohio, USA; Division of General Internal Medicine, Department of Internal Medicine, The Ohio State University College of Medicine, Columbus, Ohio, USA; Ohio Colleges of Medicine Government Resource Center, The Ohio State University, Columbus, Ohio, USA; Department of Biomedical Informatics, The Ohio State University, Columbus, Ohio, USA; Division of General Internal Medicine, Department of Internal Medicine, The Ohio State University College of Medicine, Columbus, Ohio, USA; College of Nursing, The Ohio State University, Columbus, Ohio, USA; Department of Biomedical Informatics, The Ohio State University, Columbus, Ohio, USA; Division of Maternal Fetal Medicine, Department of Obstetrics and Gynecology, The Ohio State University College of Medicine, Columbus, Ohio, USA; Division of General Internal Medicine, Department of Internal Medicine, The Ohio State University College of Medicine, Columbus, Ohio, USA; Division of General Internal Medicine, Department of Internal Medicine, The Ohio State University College of Medicine, Columbus, Ohio, USA; College of Social Work, The Ohio State University, Columbus, Ohio, USA; Ohio Colleges of Medicine Government Resource Center, The Ohio State University, Columbus, Ohio, USA

**Keywords:** maternal health services, postnatal care, electronic health records, data retrieval, primary care

## Abstract

The Multimodal Maternal Infant Perinatal Outpatient Delivery System (MOMI PODS) was developed to facilitate the pregnancy to postpartum primary care transition, particularly for individuals at risk for severe maternal morbidity, via a unique multidisciplinary model of mother/infant dyadic primary care. Specialized clinical informatics platforms are critical to ensuring the feasibility and scalability of MOMI PODS and a smooth perinatal transition into longitudinal postpartum primary care. In this manuscript, we describe the MOMI PODS transition and management clinical informatics platforms developed to facilitate MOMI PODS referrals, scheduling, evidence-based multidisciplinary care, and program evaluation. We discuss opportunities and lessons learned associated with our applied methods, as advances in clinical informatics have considerable potential to enhance the quality and evaluation of innovative maternal health programs like MOMI PODS.

## BACKGROUND AND NEED

The United States (US) is amidst a maternal health crisis with the highest maternal mortality and morbidity (MMM) rates among developed nations^1^; the US is the only developed country where rates of maternal mortality have increased since 1990.^2^ Rates of severe maternal morbidity (SMM) have increased by almost 200% over the last 3 decades, currently affecting more than 60 000 Americans annually,^3^ and disproportionately affecting minoritized racial and ethnic groups.^2^^,^^4^^,^^5^ A major driver of increasing MMM is maternal chronic illness,^3^ and there is growing recognition that addressing these conditions requires both clinical management^6^ and assessment of social determinants of health (SDoH).^7^ Solutions to address MMM must be multifaceted and tackle problems that include socioeconomic inequities, causes of MMM across the full postpartum year,^2^ low rates of postpartum visits and primary care transitions,^8^^,^^9^ and inadequacy of US health system interventions in meeting unique needs of postpartum individuals.^10^^,^^11^ Integrated models of care that ensure longitudinal postpartum care could comprehensively address the complex, yet interrelated, drivers of MMM and socioeconomic, racial, and ethnic health disparities.^12^

To address the care needs of the mother/infant dyad, we implemented the Multimodal Maternal Infant Perinatal Outpatient Delivery System (MOMI PODS), an integrated model of care that provides mom and baby with postpartum and pediatric primary care in tandem.^12–14^ MOMI PODS provides perinatal transition care, spanning multiple clinical departments and primary care partners utilizing electronic health records (EHRs) to provide care linkage, measure implementation, and track eligible and enrolled participants longitudinally.

The complexities of multidisciplinary, multisetting care accessed by individuals during the pregnancy to postpartum transition requires robust clinical informatics platforms. These platforms support integrated delivery by facilitating clinical transitions, team-based communication, and tailored care. Developing integrated data management systems are also essential for monitoring implementation and quality.^15^^,^^16^ To address the unique informatics needs of such a program, we tailored EHR tools to support MOMI PODS activities, which plays a foundational role in our ability to successfully implement, scale, and sustain the program. In this manuscript, we describe the clinical informatics processes and functions developed to implement MOMI PODS, as well as functions under development to further enhance the program.

## PROGRAM WORKFLOW

As mothers are referred by social workers, case managers, obstetricians, or other healthcare providers, they receive outreach via phone, direct mail and electronic messaging utilizing the EHR patient portal, MyChart. Mothers who accept the referral are scheduled for an appointment with a participating MOMI PODS primary care provider (PCP). Mother and infant are provided care by the same PCP and have the option of an individual or dyad visit during the child’s first 1000 days of life, after which time they are transitioned into traditional care models with the same provider. Mothers receive a comprehensive health assessment, tailored counseling, education and/or referrals to healthcare providers or community partners. Data for mother and child are entered into either csv file or patient EHR; scheduled nurse care coordinator outreaches to referred patients as well as enrolled dyads are manually tracked in csv file to delineate these from outreaches for specific needs (eg, transportation, counseling resources) or patient-initiated communication to ensure regular comprehensive assessment of family needs. The csv file is store securely on a HIPAA-compliant shared drive and managed by the nurse care coordinator. All other data, including referrals and screenings are housed within patient EHR. All data are submitted to a third-party QI data partner on a biweekly basis and linked with EHR data to track process and outcome data metrics related to clinical metrics; an example of the data report format is shown in Supplementary Figure S1. MOMI PODS was reviewed by the Ohio State University Institutional Review Board and determined to be quality improvement (QI) and not human subjects research.

### MOMI PODS informatics teams and tools

**Figure 1.**
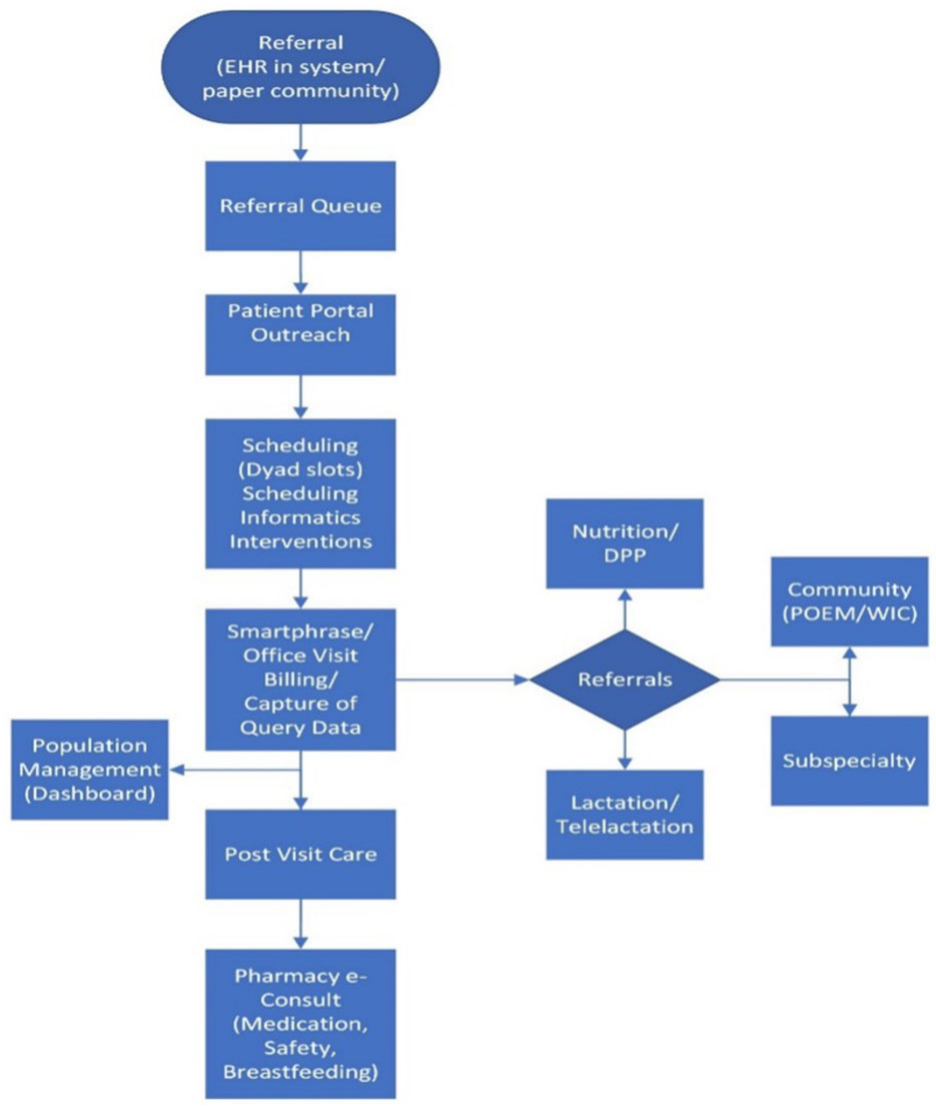
MOMI PODS informatics process.

The MOMI PODS program established a multispecialty informatics committee to build the program’s informatics platforms in parallel with the clinical program. Committee members include 2 PCPs, an obstetrician, a registered nurse, a social worker, a nurse informatician, an EHR analyst, an EHR design expert, a QI implementation expert, a bioinformatician, and 4 QI data and Medicaid claims subject matter experts. The informatics steering committee meets weekly to address current and future informatics tools, identify barriers and facilitators to utilization, determine effectiveness of data capture, develop strategies to increase clinician uptake of informatics resources, and address data gaps through EHR reporting optimization. Steering committee members also elicit program feedback from MOMI PODS clinicians and patient advisors to optimize informatics resources. The informatics steering committee collaborates with the institutional EHR prioritization committee and builders to vet and build new informatics tools in alignment with local EHR structures. The MOMI PODS informatics steering committee established a portfolio of EHR tools to support clinical processes (Figure 1).

Our institution has used the Epic EHR System for almost 15 years. MOMI PODS enrollees are referred to the program via self-referral or through the EHR system by OB/GYN providers, PCPs, local Women, Infants, and Children (WIC) sites, and Nationwide Children’s Hospital Neonatal Intensive Care Units. The referrals are received through a module in the EHR system linking the referral and patient EHR, if available. The referrals are also manually tracked by a nurse care coordinator daily using an Excel file. The spreadsheet encompasses data such as referrals, enrollment status (eg, accepted, declined), visit status (scheduled, attended, and no-show), outreach, and program completion. This format allows the nurse care coordinator to centrally track number of outreaches (eg, all referred patients receive at least 3 outreaches to determine if they are interested in enrolling prior to being marked “no response”), follow-up after a no-show appointment, and other population-based outreaches without including all other clinical interactions with the patient.

Standardized operating procedures for scheduling, rooming, and visit content have been implemented by 7 participating primary care clinics and 44 individual providers. Embedded SmartPhrase modules prompt healthcare professionals on evidence-based screening and counseling opportunities during each visit that are recorded in the EHR. In addition to outreach by the nurse care coordinator, patients receive electronic messages via MyChart that provide tailored healthy lifestyle education, program information, scheduling assistance, community resource referrals, and a survey to collect patient reported outcomes and elicit patient feedback about the program. This infrastructure has allowed collaboration across specialties, care environments, and disciplines, supporting a smooth perinatal care transition. Tailored EHR functions supporting these clinical processes that have been developed and launched are outlined in Table 1, with several initiatives in development (eg, real-time dashboard, mother/infant EHR linkage).

**Table 1.** MOMI PODS informatics infrastructure components

Function	Description
EHR Referral	Facilitates referral to MOMI PODS dyadic care Allows external tracking of referrals and enrollments Transmits patient demographics to RN care coordinator Provider facing to improve handoffs Allows for interdisciplinary communication of care needs Triggers outreach and scheduling
Templated Dyad Visit	Facilitates MOMI PODS patient scheduling Dedicated dyad slots hold space for extended visit times Allows for multiple concurrent services (eg, lactation + primary care)
MyChart Welcome Message	Facilitates MOMI PODS pregnancy to postpartum primary care transition Patient facing to improve primary care transition
Telephone Message Script	Facilitates MOMI PODS pregnancy to postpartum primary care transition Patient facing to engage in primary care transition
Staff Messaging via EHR	Facilitates evidence-based MOMI PODS dyadic care Staff facing to alert team of upcoming care Allows for pre-visit planning
SmartPhrase	Facilitates evidence-based MOMI PODS dyadic care Provider facing to alert clinician to care needs Promotes universally recommended clinical service delivery
Mood Disorder Screening	Facilitates evidence-based MOMI PODS dyadic care Promotes depression and anxiety screening and follow up
Telehealth	Facilitates evidence-based MOMI PODS dyadic care Promotes long-term follow up (eg, chronic disease management) Meets patient-specific needs (eg, telelactation, telecounseling)
eConsults	Facilitates evidence-based MOMI PODS dyadic care Promotes safety (eg, pharmacy eConsult for medication safety) Connects patients with subspecialties (eg, perinatal cardiovascular clinic)
Inter-Provider Health Information Exchange (HIE)	Facilitates multidisciplinary communication during MOMI PODS care Provider facing to alert team of tailored care needs Allows for multidisciplinary collaboration across specialties
MyChart Embedded REDCap Survey	Facilitates MOMI PODS program evaluation Collects patient-reported outcome data Collects patient feedback
Information Warehouse Data Reports	Facilitates MOMI PODS program evaluation Provides reports on preventive and emergency healthcare usage Provides reports on evidence-based patient care Provides reports on clinical outcomes Supports evaluation of implementation outcomes Supports monitoring of clinical outcomes

### Informatics-enabled program evaluation

Queries were constructed to retrieve data from the EHR relevant to the pregnancy and postpartum period for eligible patients. This included demographics, diabetes testing, depression screening, referrals for lactation consultations, postpartum visits, and others. Queries were also written for infant data, including immunizations and well child visits. Data from the EHR were supplemented by data from vital statistics (VS) records and Medicaid claims.

Probabilistic linkage was used to match records from the EHR, VS, and Medicaid claims. This process was chosen due to limited availability of unique identifiers across all 3 data sources. A combination of nonunique identifiers such as first name, last name, date of birth, and county of residence, is used to match records from one data source to another. First, 2 probabilities are estimated for every identifier: an M-probability and a U-probability. These probabilities are estimated empirically using the expectation-maximization algorithm.^17^ The result of the linkage process is the ability to identify care provided at other facilities. When a birth occurs outside of the health system, EHR query has difficulty linking the infant to the mother, however linkage to VS allows identification of births outside of our health system—we have found 13 births in VS (∼10% of our infant participants) that were not identified in the EHR extract. Claims data will find care at any facility when Medicaid is billed, thus allows comprehensive data capture for patients with public insurance. We found 12 mothers (10% of the adult participants) who had ER visits outside of the health system that would have been missed without Medicaid data linkage. When no link is found to VS or Medicaid eligibility files, we can lower the threshold of the calculated linkage weights that define a match, but this rarely results in additional matches found. Lack of matches in VS were most often related to a lag in the file being updated/birth occurring outside of Ohio.

Next, we compared the values for the identifiers for pairs of records between data. To reduce the number record pairs for consideration, blocking was used. After comparing the values for each identifier, a matching score is calculated. Data from the EHR, VS, and Medicaid claims were merged and analyzed using R statistical software.

### Opportunities and lessons learned

As of February 2023, 253 referrals have been received and 222 patients enrolled including 125 mothers and 97 children. Data of mother and child for EHR had a 95% linkage rate, VS had an 86% linkage rate and Medicaid claims had a 68% linkage rate. A lesson learned was that linking dyads to claims data proved challenging, due to the lack of Medicaid IDs in our EHR. To overcome this, we needed to use probabilistic linkage methods in order to maximize the number of mothers found in the claims eligibility files. More than half of the matches we found (60%) had an EHR record with no Medicaid ID or one that did not match the ID in the eligibility file. The reports were created at the collaborative and site level and shared monthly with participating clinics. Report domains summarized monthly enrollment, demographics, maternal health behaviors, mental health screenings, rates of chronic conditions (eg, hypertension), and infant health parameters (eg, vaccination rates). Data were longitudinal and covered all health system engagement from the beginning of pregnancy through the duration of the MOMI PODS program. Data reports were used by sites to identify deviation of patient care from clinical best practice, patient engagement in the MOMI PODS program, and track clinical outcomes. Preliminary MOMI PODS implementation has demonstrated high rates of postpartum healthcare service delivery, chronic disease management,^18^ screening, and treatment of mood disorders,^18^^,^^19^ accompanied by widespread health system engagement and program adoption.

There were also some key barriers encountered by the MOMI PODS sites. First, the data extraction and analysis process relied on consistent input from clinical partners, which was imperfect. Thus, the MOMI PODS team engaged partners proactively to define a shared understanding of data elements, definitions, and interval frequencies to bolster accurate data reporting. One of the key lessons learned was the importance of engaging a multidisciplinary data team early in program development. EHR data experts were able to identify areas of the EHR that allowed for consistent data extraction; unfortunately, this does not include clinician notes, where many variables we were interested in tracking were recorded in the initial stage of MOMI PODS. Due to this, a new evaluation plan was developed via a collaborative informatics and clinician team. Clinician stakeholders were able to work with informatics experts to identify sources in EHR that were not only mineable but also likely to be utilized in existing clinical practice. Clinician data team members would then work to encourage increased uptake of new documentation practices amongst their staff and colleagues. Additionally, MOMI PODS sites were tasked with utilizing consistent clinical documentation across sites which required ongoing training and communication between the informatics and healthcare teams to balance program evaluation with clinical feasibility. Accounting for ongoing support and resources that can be addressed overtime is important in consideration of the resources required for creation of a similar program. To address this, the team is developing peer-led learning sessions which will be recorded to allow for continued learning as new staff are onboarded to program activities, as well as creating a centralized shared site for all resources and clinic guides.

## DISCUSSION

MOMI PODS is an example of how EHR-integrated informatics infrastructure can play a critical role in supporting tailored perinatal care transition, postpartum primary care, and dyadic healthcare, as well as multidisciplinary communication ensuring coordinated delivery of services in a population at high risk for SMM. Despite the long-standing use of Epic Systems at our institution, limited infrastructure supporting tailored perinatal care activities existed prior to launch of the program. We have learned that establishment of the required EHR infrastructure not only requires significant time investment, but also requires the deployment of multilayered staffing and expertise, such as clinical IT staff, clinicians, administrators, and leadership steering committees. Alignment of strategic perinatal care and quality goals across multiple domains of the institution served as a prerequisite to development of MOMI PODS-specific EHR tools. With the passage of Postpartum Coverage Extension in the American Rescue Plan Act of 2021, states are now provided options for lengthening postpartum Medicaid coverage eligibility from 60-day postpartum to the full year postpartum.^20^^,^^21^ As such, now is the time to establish structures that allow for the sustained implementation of innovative and integrated models of perinatal primary care.

As we have learned through the need for ongoing adjustment of data capture and program evaluation, EHR tools are not static, but instead present opportunities for refinement in response to ongoing program changes and local clinical workflows. This responsiveness of EHR tools to changing needs ensures the ongoing relevance to the program during expansion and sustainment. When possible, building upon existing EHR tools (such as e-consults) has remained central to ensuring the ongoing adaptability of the MOMI PODS EHR tools. Deploying variations of tools commonly used by clinicians and staff in other clinical duties also streamlines the training and initiation energy for uptake. These approaches to EHR infrastructure implementation have allowed the MOMI PODS EHR tools to support implementation and collaboration of professionals across specialties and care environments. It has also facilitated clinician and geographic breadth of postpartum primary care delivery within the program. Further expansion is planned with facilitated rollout of the MOMI PODS system wide transition care program spanning multiple departments/sites with an aspiration to span the entire central Ohio region.

These established EHR tools allow the MOMI PODS team to implement data-driven QI processes, address gaps in care, and increase delivery of evidence-based postpartum interventions. Furthermore, EHR data collection from these tools, when linked to Medicaid claims data and VS data, allows for a robust longitudinal evaluation which addresses prescription fill rates, patients’ completion of specialty referrals, utilization of externally affiliated health care systems, and clinical outcomes, essential to demonstrating program value for sustaining support. Despite these EHR-driven successes in program evaluation, more work is needed in parent-baby EHR linkage and SDoH capture to optimize clinical processes, service delivery, data gathering, and evaluation.

## CONCLUSION

Investment of resources to establish tailored clinical informatics tools for MOMI PODS perinatal care components has proven foundational to the program’s clinical service delivery, multidisciplinary healthcare communication, QI and implementation initiatives, and program evaluation. We have learned that organizations seeking to establish similar programs must prioritize and institutional alignment and multidisciplinary mobilization of informatics and clinician teams to establish the EHR tools that fuel success of the programs. National policies and funding priorities supporting the tailoring of informatics technologies to address women’s unique healthcare can fuel the rapid address of informatics gaps and rapid deployment of clinical innovation to improve maternal child health outcomes across the pregnancy continuum.

## Supplementary Material

ooad065_Supplementary_Data

## Data Availability

The institutional deidentified data underlying this article are available from the corresponding author on reasonable request and institutional approval. Interested parties will be required to complete an institutional Data Use Agreement, and data will be made available via Secure Data transfer after institutional approval is secured.
