# Supplementary material for: Establishing a clinical informatics umbilical cord: lessons learned in launching infrastructure to support dyadic mother/infant primary care
Source: JAMIA Open. 2023 Aug 18;6(3):ooad065. doi: 10.1093/jamiaopen/ooad065 (PMC10438959; doi:10.1093/jamiaopen/ooad065)
Supplement: ooad065_Supplementary_Data [file ooad065_supplementary_data.pdf]

Sample of Items from MOMI PODS Monthly Report

MOMI PODS Monthly Report - April 2023

Monthly enrollment
Demographics - mom
Health behavior - mom
Mental health
Hypertension
Diabetes
Infants

This report is based on data refreshed on 2023-05-01.

Month	Referred	Contacted	Enrolled	Declined	Withdrawn	Cumulative enrolled	Cumulative withdrawn	Total in study this month (Cumulative enrolled - withdrawn)
2021	98	98	12	21	0	12	0	12
January-2022	19	13	17	0	0	29	0	29
February-2022	44	44	20	13	0	49	0	49
March-2022	85	85	46	10	0	95	0	95
April-2022	74	74	71	15	0	166	0	166
May-2022	48	40	31	0	4	197	4	193
June-2022	87	95	54	0	3	251	7	244
July-2022	108	108	78	8	19	329	26	303
August-2022	403	400	143	20	0	472	26	446
September-2022	208	211	124	18	20	596	46	550
October-2022	152	148	50	16	37	646	83	563
November-2022	117	117	96	0	25	742	108	634
December-2022	98	107	79	0	10	821	118	703
January-2023	195	180	127	32	31	948	149	799
February-2023	90	80	83	6	15	1031	164	867
March-2023	78	76	25	0	28	1056	192	864
April-2023	74	72	16	0	0	1072	192	880
Total	1978	1948	1072	159	192			

Note: In this report, EHR data is available for:

- **790** moms
- **680** infants (600 unique moms)

Vital statistics records were matched to:

- **600** infants from the EHR plus
- **40** infants were in VS but not EHR. Demographic info for these infants are included in report.

Medicaid claims matched to:

- **474** moms
- **360** infants

Figure 1. Monthly enrollment table

MOMI PODS Monthly Report - April 2023

- Monthly enrollment
- Demographics - mom
- Health behavior - mom
- Mental health
- Hypertension
- Diabetes
- Infants

	Overall (N=1000)
**Age at enrollment**	
Mean (SD)	29.7 (5.8)
Range	18.0 - 45.0
**Age, categorized**	
1) 18-24	204 (20.4%)
2) 25-34	591 (59.1%)
3) 35-44	197 (19.7%)
4) 45+	8 (0.8%)
**Ethnicity**	
Hispanic	186 (18.8%)
Non-Hispanic	806 (81.2%)
N-Miss	8
**Race**	
Non-Hispanic White	336 (33.9%)
Non-Hispanic Black/African American	348 (35.1%)
Asian/Pacific Islander	53 (5.3%)
Other	255 (25.7%)
N-Miss	8
**County**	
Franklin	786 (87.5%)
Licking	39 (4.3%)
Other	73 (8.1%)
N-Miss	102
**Pregnancy status**	
Pregnant	108 (10.8%)
Postpartum	892 (89.2%)
**Mother's education**	
1) 4-yr college degree or higher	292 (35.1%)
2) Associate degree	105 (12.6%)
3) Some college	105 (12.6%)
4) High school diploma or equivalent	263 (31.6%)
5) No high school diploma	67 (8.1%)
N-Miss	168
**Insurance**	
1) Medicaid	376 (44.4%)
2) Not Medicaid	470 (55.6%)
N-Miss	154

Figure 2. Demographics

MOMI PODS Monthly Report - April 2023

- Monthly enrollment
- Demographics - mom
- Health behavior - mom
- Mental health
- Hypertension
- Diabetes
- Infants

▼ ER visits

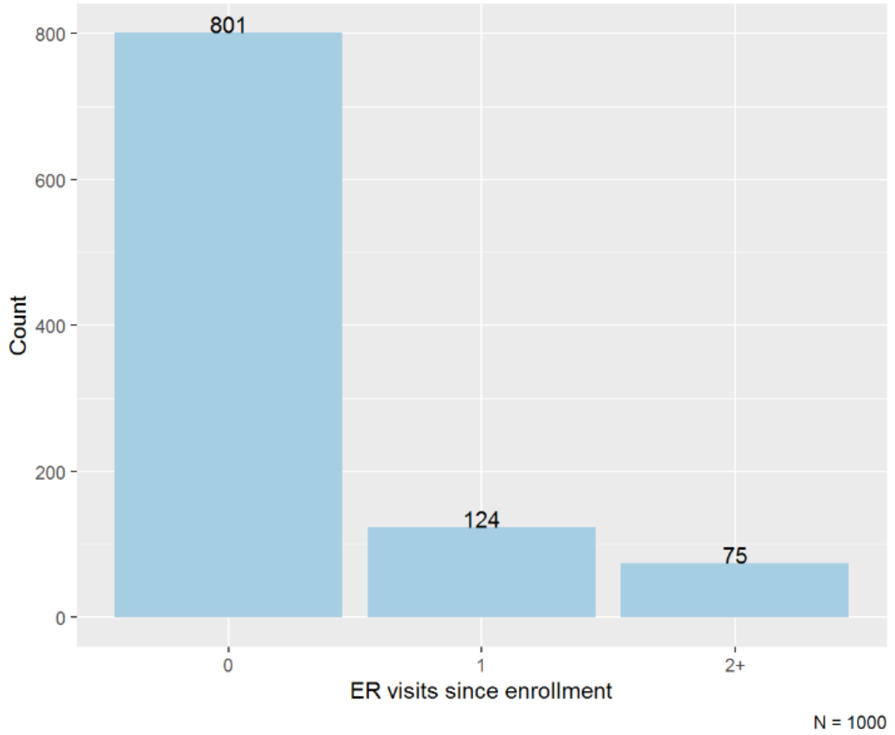

- ▶ Weeks gestation at 1st prenatal visit among those that are postpartum
- ▶ Number of prenatal visits among those that are postpartum
- ▶ Registered dietician, cumulative number of all patients with referral
- ▶ Registered dietitian, cumulative number of all patients with referral, among patients with GDM
- ▶ Average number of months between last and current pregnancy, among postpartum
- ▶ STD screening, cumulative number screened
- ▶ MOMI POD postpartum enrollees ever attended a postpartum visit
- ▶ Additional Health Behaviors of the Mother

Figure 3. Number of visits to the emergency department among MOMI PODS enrollees

MOMI PODS Monthly Report - April 2023

Monthly enrollment

Demographics - mom

Health behavior - mom

Mental health

Hypertension

Diabetes

Infants

▼ Screening for depression (PHQ or EPDS) and follow-up

Screening for depression

Name	Numerator	N	Percent
Screening For Depression Since 12 Months of Data Update for This Report	909	1000	90.9
Screening For Depression By 13 Weeks Postpartum Among Postpartum Women	681	753	90.4

▶ Screening for Anxiety (GAD)

Figure 5. Percent of MOMI PODS enrollees screened for depression
